# Oriented cell division: new roles in guiding skin wound repair and regeneration

**DOI:** 10.1042/BSR20150225

**Published:** 2015-12-24

**Authors:** Shaowei Yang, Kui Ma, Zhijun Geng, Xiaoyan Sun, Xiaobing Fu

**Affiliations:** * Wound Healing and Cell Biology Laboratory, Institute of Basic Medical Science, Trauma Center of Postgraduate Medical School, Chinese PLA General Hospital, 28 Fu Xing Road, Beijing 100853, P. R. China; † Key Research Laboratory of Tissue Repair and Regeneration of PLA, and Beijing Key Research Laboratory of Skin Injury, Repair and Regeneration, First Affiliated Hospital to the Chinese PLA General Hospital, 51 Fu Cheng Road, Beijing 100048, P.R. China

**Keywords:** division axis, oriented cell division, spindle orientation, wound healing

## Abstract

Tissue morphogenesis depends on precise regulation and timely co-ordination of cell division and also on the control of the direction of cell division. Establishment of polarity division axis, correct alignment of the mitotic spindle, segregation of fate determinants equally or unequally between daughter cells, are essential for the realization of oriented cell division. Furthermore, oriented cell division is regulated by intrinsic cues, extrinsic cues and other cues, such as cell geometry and polarity. However, dysregulation of cell division orientation could lead to abnormal tissue development and function. In the present study, we review recent studies on the molecular mechanism of cell division orientation and explain their new roles in skin repair and regeneration.

## Abstract (precis)

In the present review, we first present an overview of oriented cell division process and related proteins and molecular signals in different developmental contexts. We speculate that oriented cell division involves in epithelial polarization and skin wound healing.

## INTRODUCTION

Tissues develop in a precisely controlled course to produce adult structures with the correct size and shape. Much progress has been made in understanding the molecular pathways that control tissue size [1–3]. However, much less is known about the mechanisms that control tissue shape. Recent evidence indicates that the orientation of the cell division plane plays an important role in co-ordinating division rate with cell fate choices and cell position, which further specifies the repertoire of cell types, the organizational structure and shape of a tissue or organ.

Oriented cell division mainly depends on the location of the mitotic spindle. Through most of the mitosis cycle, the spindle apparatus has no fixed structure and is constantly changing. However, during metaphase, the mitotic spindle forms two spindle poles constructed with three types of microtubules: kinetochore microtubules which attach to chromosomes; astral microtubules that radiate out from spindle poles and anchor the mitotic spindle to the cytoplasm and cell cortex; and interpolar microtubules that form an antiparallel array between the spindle poles [4,5]. Proper location and direction of the mitotic spindle apparatus requires pulling forces acting on astral microtubules [6,7]. Although the regulation of microtubule dynamic instability is necessary for correct spindle positioning, the alignment of the spindle relies on the interactions of astral microtubules with the cell cortex and cytoplasmic anchor sites [8]. Cell–cell contacts and cell adhesion to their extracellular matrix (ECM) is also a key determinant of mitotic spindle orientation. Meanwhile, tissue morphogenesis is determined by the precise balance between asymmetric and symmetric cell divisions. Asymmetric cell divisions generate cellular diversity, whereas symmetric cell divisions expand the pool of similar cells [9]. Spindle orientation couples intrinsic cues of polarity, but also locates relative to cell-extrinsic cues such as niche-derived signals, which affects the choice of symmetric or asymmetric cell fates. Thus, dysregulation of oriented cell division could result in abnormal development and function of the tissue and cell fate mis-specification.

In the present review, we first present an overview of oriented cell division process in different developmental contexts. Then, we summarize a panel of proteins and molecular signals involved in orienting cell division. We speculate that facultative regulation of symmetric or asymmetric divisions by targeting the molecules responsible for oriented cell division, focusing specifically on epithelial polarization, may provide a new alternative to improve adult regenerative capacity in the skin during wound healing.

## ORIENTED CELL DIVISION IN THE DEVELOPMENT OF ORGANISMS

All metazoans are faced with two basic developmental challenges: generating cellular diversity and shaping tissue morphology. Oriented cell division regulates embryogenesis, organogenesis and cellular differentiation and functions from a single cell zygote to the adult tissues and organs.

### Shaping tissues and organs

Oriented cell divisions could drive morphogenesis by shaping tissues. These include the elongation of tissues, branching morphogenesis and maintenance of single lumens within epithelial tubes. Oriented cell division during growth and development has been studied in great detail and to great effect in *Drosophila*.

During early *Drosophila melanogaster* embryogenesis, the germ band extends and elongates along the anterior–posterior axis of the embryo after gastrulation. This process requires both cell intercalations and oriented cell divisions. In relation to the long axis of the extending tissue, cell division is executed preferentially along the anterior–posterior axis. Tissue elongation is not completely prevented by blocking cell divisions, but the amount of extension is reduced. Moreover, randomized spindle orientation causes an isotropic increase in tissue size in mutant embryos on account of lacking segmentation pattern [10]. Oriented cell division is involved in germ band extension. However, it is difficult to specify its individual role in the process because of the overlap between cell division and cell intercalations. Later in development, measurement of spindle orientation in the wing blade and in the eye disc further verifies that cell division plays a greater role than cell relocation in defining clonal shape [11,12].

Another example of oriented cell divisions in morphogenesis occurs in zebrafish. During gastrulation, the majority of cell divisions are oriented along the animal–vegetal axis in the dorsal region of the midline and later in the ventral region of the epiblast surface layer [13–15]. Similarly, during neurulation, most of neural plate cells undergo midline-crossing divisions [16]. The mitotic spindle rotates 90 degrees in the oriented cell division across the midline [17]. Cell division is essential for these midline crossing events, as blocking cell division prevents most cells from crossing the midline [18,19]. But proper orientation of these divisions is required, as perturbation of oriented cell divisions in the neuroepithelium results in severe defects in the neural rod midline [13,20]. Even unicellular organisms, such as yeast, exploit spindle orientation to grow as hyphae [21,22].

### Generating cellular diversity

In addition to shaping tissues and organs, oriented cell division can generate cellular diversity, which mainly involves asymmetric cell division [23]. This is achieved by unequal partitioning of cell fate determinants and into asymmetrical daughter cells, which is required for the proper alignment of the mitotic spindle corresponding to an internal or external polarity axis. Studies performed in model organisms such as the early *Caenorhabditis elegans* embryo [24–26], *Drosophila* neuroblasts [23] and *Drosophila* sensory organ precursor (SOP) cells [27], have made great contributions in understanding asymmetric cell division.

Known as neural stem cell-like cells, *Drosophila* neuroblasts orient their mitotic spindle along an established axis of internal polarity. Segregation of the cell fate determinants asymmetrically into two daughter cells, depends upon the stereotypical spindle orientation. One is a small differentiating ganglion mother cell (GMC), the other is an apical neuroblast retaining self-renewed [10,28]. Cell dissociation experiments in *C. elegans* show that embryonic neuroblasts related neuroepithelial cells divide along a stabilized division axis over successive rounds of divisions, whereas unrelated neuroblasts divide along random division axes, which reveals that unknown extrinsic factors are essential for maintaining correct neuroblasts division orientation in the fly embryo, other than neuroblasts intrinsic polarity cues [29].

Likewise, soon after fertilization, the proper orientation of the mitotic spindle is required to the polarized early *C. elegans* embryo firstly. Spindle orientation and displacement then proceeds in two stages: first, the nuclear–centrosome complex moves to the centre of the cell and rotates 90° during prophase; second, the spindle is pulled to the posterior of cell during metaphase and anaphase. Finishing the process requires not only the interactions between the mitotic spindle and the cortex, but also the intrinsic polarity cues [30,31]. The proper segregation of cell fate determinants is also essential, which needs the appropriate spindle orientation [28]. These studies illustrate that oriented cell division could control the organ development as a ubiquitous morphogenetic manner in a variety of species.

## MECHANISMS OF SPINDLE ORIENTATION IN CELL DIVISION

Since oriented cell division is critical to the development and growth of the organism and the division axis is a basic regulator in cell division, what are the cellular and molecular determinants modulating correct cell division orientation and how do these determinants differ among the different cell types and species?

In metazoan development, oriented cell division depends upon the mitotic spindle orientation. However, spindle orientation can be controlled by intrinsic and external cues. Intrinsic cues rely on the partitioning of cell components that determine cell fate. External cues involve the placement of daughter cells associated with external cues. Furthermore, physical constraints, such as cellular environment and cell geometry, may influence cell division orientation [32,33].

### Extrinsic cues

**Figure 1:**
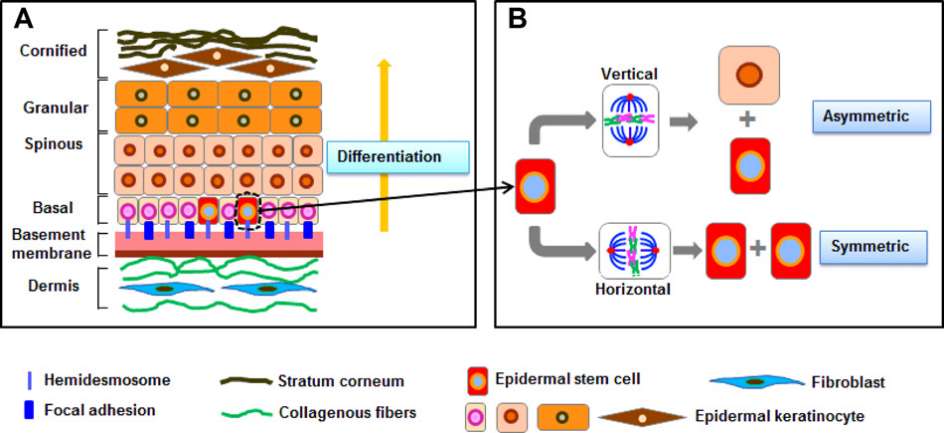
Extrinsic and intrinsic polarity cues in oriented cell division (**A**) In *Drosophila*, divisions of SOP cells occur along the anterior–posterior axis. (**B**) Before divisions of SOP cells, centrosome duplication and nuclear envelope breakdown occur. Centrosome migration and spindle rotation align mitotic spindle to establish an in an axis. The core PCP pathway mainly participates in the extrinsic cues. The transmembrane protein receptor Fz and its cortical effector Dsh localize to the posterior cortex, which restricts Fz signalling and ultimately translates into planar polarization. Another PCP protein, Stbm, localizes to the anterior cortex and recruits Pins, where they both work to restrict partitioning defective protein (Par) 3/6 to the posterior cortex. (**C**) The Gα–Pins–Mud pathway (Gα–LGN–NuMA in mammals) takes part in the intrinsic cues in *Drosophila*. The Insc co-localizes with Par3/6 and forms an apical crescent at the cortex in *Drosophila* neuroblasts. Then, Insc recruits Pins which could recruit Mud and this interaction provides a link between the polarity cues and the mitotic spindle. The Gα–Pins–Mud complex recruit Mud to the apical cortex Asymmetric location of Gα, Pins/LGN and Mud/NuMA induces spindle orientation in *Drosophila* neuroblasts and mammal basal cells. And the two complexes are respectively binding with Par3–Par6–aPKC via Insc. Besides, the dynein complex acts as a force generator.

*Drosophila* SOP cells use extrinsic signals to establish their anterior–posterior axis of division (Figures 1A and 1B). The core planar cell polarity (PCP) pathway is used to define this axis within the epithelium plane. The seven-pass transmembrane protein receptor frizzled (Fz) and its cortical effector dishevelled (Dsh) localize to the posterior cortex of the SOP cell. This localization restricts Fz signalling to the posterior of the cell, which ultimately translates into planar polarization. At the same time, another PCP protein, Strabismus (Stbm), localizes to the anterior cortex and recruits partner of Insc (inscuteable; Pins), where they both work to restrict partitioning defective protein (Par) 3/6 to the posterior cortex [34,35]. Like the *C. elegans* zygote, because of lacking Insc expression in SOP cells, Pins and the Par proteins localize to opposite sides of the cell. Interestingly, spindle orientation along the anterior–posterior axis does not require Pins or the Par complex. Instead, both Fz and Dsh have been shown to be required [34,36]. This is possibly due to the binding of Dsh to mushroom body defect (Mud), linking extrinsic signalling through the Fz pathway to regulation of the mitotic spindle [37]. Oriented cell divisions during zebrafish gastrulation have been shown to use this pathway as well [14].

During zebrafish gastrulation, epiblast cells generate the neural ectoderm on the dorsal side of the epidermis [14,38,39]. Inhibition of Dsh could lead to defective polarity of elongation and randomization of cell division orientation in all epiblast layers [17]. Dsh participates in the canonical Wnt/β-catenin pathway and PCP pathway. Inhibiting classical Wnt signalling did not affect oriented cell division. Oriented cell division is not influenced by inhibiting classical Wnt signalling, but by blocking the two PCP ligands Wnt11 or Wnt5 [17]. Furthermore, lacking Fz7 (receptor for Wnt11) [40] and Stbm show randomized spindle orientation in zebrafish [13,17].

Addition to relying on Fz and Dsh, Fat and Daschsous can also regulate the PCP pathway. For example, in the *Drosophila* wing, proper oriented cell divisions require Fat and Daschsous [11]. A gradient of Daschsous is expressed along the proximal-distal axis of the wing [41]. Daschsous can bind to Fat and transduce the signal to atypical myosin Dachs. In response to the signal, Dachs becomes localized distally [42]. Mutant clones of Fat, Daschsous or Dachs show defects in spindle orientation [11]. Additionally, Daschsous is sufficient to induce oriented cell divisions in the wing, because the direction of cell division alters as the direction of Daschsous' gradient alters [12]. These studies demonstrate that PCP and Ft/Dsh signalling system conducting extrinsic cues plays part in oriented cell division.

### Intrinsic cues

Cell intrinsic polarity mainly functions in asymmetric cell division. Par/Insc pathway plays a crucial role in intrinsic polarity of cell division orientation.

*Drosophila* neuroblasts have the ability to orient their mitotic spindle even in absence of extrinsic cues. When cultured and removed from their normal environment, mitotic neuroblasts were still able to localize the key components required for spindle orientation. Furthermore, the resulting asymmetric division produces one neuroblast and one GMC [43]. Other than extrinsic cues, the intrinsic polarization of the neuroblasts is required to refine the process. Only when in contact with epithelial cells are the neuroblasts able to maintain proper positioning of the centrosome and polarize the Par complex at prophase [29]. The initial polarity of these cells comes from the polarized neuroectoderm from which the neuroblasts are derived. In the neuroectoderm, the polarity proteins Par3/6 are localized apically just above the adhesion junction. Neuroblasts then delaminate from the epithelium and inherit their polarity cues in this process. Par-3/6 in the stalk can extend into the epithelium as delamination occurs [44]. As a result, a specified neuroblast cell is formed with a clear apical–basal axis along which the spindle can align.

How does a mitotic spindle align along a predetermined polarity axis? A core component that connects the polarity cues to the mitotic spindle in the neuroblasts is the protein Insc. Insc is not expressed in the neuroectoderm, but during neuroblast delamination, Insc co-localizes with Par3/6 in the stalk. When the stalk is retracted and delamination is complete, Insc forms an apical crescent at the cortex [45]. This localization is achieved through Insc directly binding to Par3. In neuroblasts lacking Par3, Insc remains cytoplasmic and is not recruited to the apical cortex [46]. Once at the cortex, Insc recruits Pins, which is in an inactive during interphase, to the membrane during mitosis [47–49]. At the apical cortex, Pins is able to efficiently recruit Mud [50]. Interestingly, after their initial recruitment, both Insc and Pins are required to maintain each other localization at the apical cortex [47]. This range of interactions and the crystal structures of some of the interactions are clear; however, neither the molecular players nor their dynamic interactions throughout the spindle orientation process have been solved.

Moreover, the recruitment of Mud by Pins provides a link between the polarity cues and the mitotic spindle (Figure 1C). This interaction resulted in a Gα–Pins–Mud complex (homology of a Gα–LGN–NuMA complex in mammals) to recruit Mud to the apical cortex [51–53]. However, previous structural and biochemical data have clearly demonstrated that isolated LGN-binding domains of Insc and nuclear mitotic apparatus protein (NuMA) compete with one another for binding to LGN [54]. This suggests that they exist in two distinct complexes required for recruitment to the apical cortex. However, it also shows that either in the context of the full-length proteins or in the presence of additional factors, could exist in a single complex. Thus, the regulation of binding partners would not affect the integrity/localization of the complex, but could affect its activity.

Evidence indicates that the complex is able to interact with members of the dynein complex in the *C. elegans* zygote (Figure 1C). In accord with this, pulling forces can be reduced by various mutations resulting in an inactive dynein complex [55]. In addition to molecular motors, microtubule dynamics themselves can generate forces [56]. Thus a combination of forces from dynein motors and microtubule depolymerization may control spindle positioning. Therefore, it will be important to determine how these work together and/or are co-ordinated with each other.

### Other cues on spindle orientation

Over 120 years ago, Oscar Hertwig recognized that cells divide along their long cell axis, which known as the ‘long axis rule’ [57]. According to the rule, through determining the long axis and the centre of the cell, cell geometry maybe one of cues for spindle orientation. Next studies verify the observation in a variety of systems [32,33]. In normal rat kidney cells, spindle positioning and orientation are preferentially along the long axis of the cell. Once cell shapes are altered, spindle will re-orient along the new long axis of the cell, which rely on in part microtubule. Experiment shows that inhibition of dynein enriched in astral microtubules could cause the spindle to deviate from the long axis of cell, which suggests that the spindle positioning and orientation depend on in part the interactions between astral microtubules and cortical factors [58].

Many studies indicate that cell geometry and ECM is essential for the spindle orientation [18,59,60]. Temporal and spatial coherence of cell adhesion and division appeared vital characteristics of tissue homoeostasis [61]. Cell geometry reflects the spatial structure of internal forces controlled by focal adhesions and actin fibres, which correspond to external cues from the ECM or from the neighbour cells. For embryo development and tissue integrity, cell division provides steadily a continuity of the traction forces [62]. Localization of adhesion related cues on the mitotic cell cortex ensures correct the alignment of mitotic spindle, which guarantees reset the traction forces in daughter cells [63]. The intimate relationship between cell adhesion and division make orientation of the division axis correctly with respect to other cues and then make the two daughter cells inherit the adhesion pattern equally from the mother cell and help to restore mechanical integrity of the tissue controlling the shapes of cells and tissues, which regulate the cell division orientation.

In conclusion, spindle orientation requires intrinsic cues, extrinsic cues and other cues, such as cell polarity, cell geometry, cell–cell adhesion and cell–ECM adhesion. But how these cues translate into correct spindle orientation is not well understood. Particularly, the cues link between the spindle and adhesive molecular determinants should be elucidated in future studies.

## ORIENTED CELL DIVISION IN SKIN

**Figure 2:**
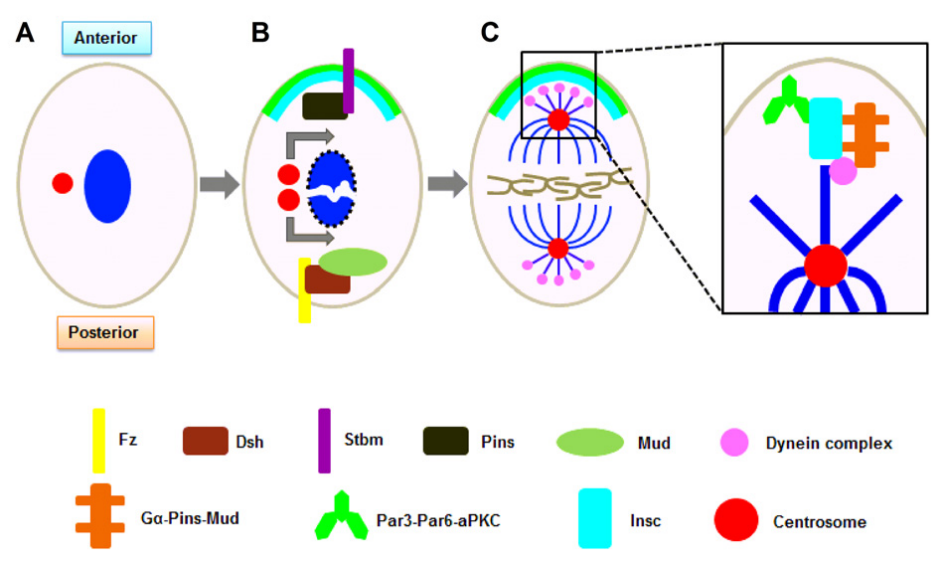
Stratification of the epidermis and oriented division of the ESCs (**A**) In normal skin, basement membrane separates the epidermis and dermis and functions as a critical regulator in the cellular and molecular communication between dermal fibroblasts and epidermal keratinocytes. ESCs in the basal layer could detach from their underlying basal lamina, they stop dividing, commit to terminal differentiate, move outward and eventually are sloughed from the skin surface. (**B**) ESCs are capable of symmetric division according to the horizontal direction of basement membrane and asymmetric division according to the vertical direction. Under normal circumstances, ESCs are in a quiescent state and only a few ESCs conduct cell division to keep self-renewal and produce daughter cells [(transit amplify cells (TAs)] that undergo differentiation. Once injured in skin, ESCs resided in the bulge region of the HFs IFE sebaceous gland and the upper isthmus region of the HFs could migrate from the wound edge to conduct asymmetric division to resurface the wound area and re-epithelialization. One of the daughter cells remains in a niche as a stem cell for future use, whereas the other daughter cell is TAs for current use. Oriented division of ESCs plays a key role in keeping the self-renewal of epidermis and especially in skin repair and regeneration.

As the outermost layer of the body, the stratified mammalian skin is consist of epidermis and dermis, which distinguished by the basement membrane [64]. The epidermis is mainly composed of basal layer, spinous layer, granular layer and cornified envelope (Figure 2A) and acts as a barrier function to prevent the physical and chemical damage and retain essential fluids. Throughout life, the epidermis must constantly keep self-renewal for homoeostasis, in which cells from a single inner basal layer periodically withdraw from the cell cycle, undergo differentiating and move upward.

### Stratification of the epidermis

Morphological studies show that early basal epidermal progenitors reorient their mitotic spindles, as they begin to develop tissue. From early embryo period to birth, most of mitotic spindles orient perpendicular rather than parallel relative to the underlying basement membrane in the epidermis [64–66]. But, perpendicular spindle orientation in the back skin of adult mice has not been observed after birth [67]. A few divisions in suprabasal located to the first suprabasal layer, which is on the account of transient window of early embryonic epidermal stratification. Both daughter cells of such divisions seem be prepare for differentiation [64]. The basal progenitors are the source of basal and suprabasal cells. On one hand, some basal cells divide symmetrically to generate daughter cells and few of which result in eventual delamination by reason of surrounding microenvironment. On the other hand, some basal cells undergo asymmetric division and one of daughter cells inherits more progenitor factors, whereas the other receives more preferential differentiating factors for delamination.

However, the choice between an asymmetric and symmetric division of basal cells is somewhat random in nature, which adjusts by the needs of the dynamic skin epithelium [66–68]. Thus, understanding how a single cell type choose can elucidate the molecular mechanisms that orient divisions in a particular tissue and how to change artificially the cell division orientation during skin wound healing to achieve the perfect tissue repair.

### Apical–basal polarity and related molecules

We have discussed the possibilities of the mechanism of oriented cell division in *Drosophila* SOP cells and neuroblasts, zebrafish gastrulation and *C. elegans* zygote. The main signalling pathways involved are Par/Insc and PCP pathway. Which of these pathways involve in orienting the spindle apical–basally in epidermis?

Several lines of genetic evidence and localization assays have suggested that Par3, Par6, atypical protein kinase C (aPKC), LGN (a genuine Pins homologue) and adaptor protein mInscuteable (mInsc) play a key role in recruiting Par3/aPKC, the apical crescent of LGN and mInsc during epidermal stratification and differentiation [64–66]. Perpendicular spindle orientation depends upon Gα, LGN, NuMA and dynactin 1 (Dctn1) by localization assays. However, mammalian LGN isoform is required for asymmetric cell divisions in neural progenitors, is not necessary for that in epidermis [65,69].

Whereas the role of LGN and NuMA in asymmetric divisions is clear, we still have only a few minor hints of the role of mInsc. At least, mInsc could establish a direct relationship with the cortical polarity by interacting with Par3, although how it functions remains unknown [70–72]. Experiment showed that alignment of NuMA in the cell cortex position for locating the spindle apical–basally requires uncoupling of mInsc [50]. However, NuMA does not bind to mInsc directly and its interaction with LGN is mutually exclusive from the binding of mInsc to LGN. Thus, it is need to be answered how the Gα–LGN–NuMA pathway interacts with cortical Par3–mInsc–LGN during spindle orientation. In addition, discs large (Dlg) may function in regulating apical polarity complexes in mammalian epithelial tissues, whereas the exact molecular mechanism needs to be further clarified [65,73–76].

### Asymmetric compared with symmetric divisions: how to choose?

Significant progress has been made in understanding the issue of the positioning of orientation in asymmetric divisions; however, how spindles in lateral divisions become oriented and switch to perpendicular spindle orientation is not fully understood. Then, what are the factors influencing oriented cell division in the skin.

As stated above, centrosome migration and spindle rotation play the key role in oriented cell division. Unlike other tissues, both symmetric and asymmetric cell divisions occur in the stratifying epidermis. Many studies have verified that spindle orientations could be either in perpendicular or in parallel to the basement membrane, which respectively corresponded to asymmetric and symmetric cell division, in the interfollicular epidermis (IFE) [77], hair follicles (HFs) [78,79] and in sebaceous gland development [80], where the epidermal stem cells (ESCs) reside. The balance between asymmetric and symmetric cell division is required for increasing the surface area of the epidermis for covering the body and generating the required differentiated layers for a right thickness of epidermis. ESCs in the skin divide symmetrically and a transit amplifying cell divides asymmetrically. The different proliferation rates of each cell type could be regulated as various cell types were needed. Every epidermal progenitor is equivalent and has the ability to divide in either orientation [66], which indicates that the microenvironment plays an important role in the overall outcome of cell divisions. For example, changes of the adhesive cues could result in a random spindle orientation that leads to altered differentiation patterns in mouse epidermis [64]. Therefore, the ratio of asymmetric/symmetric cell division would be the sum of individual cell choices. Besides, the switch from predominant symmetric divisions to asymmetric divisions coincides with the onset of stratification, indicating that asymmetric cell division promotes epidermal differentiation.

Study has shown that depletion of NuMA, LGN and Dctn1 causes a bias towards lateral spindle orientation in planar analyses, which indicates that 2D spindle orientation may be an abnormal pathway with respect to the original cues [65]. During a perpendicularly oriented division, NuMA presents at the apical pole of dividing cells, but enriched at both spindle poles in most lateral divisions, suggesting that it is also insufficient in expression and stability of asymmetric division machinery itself [64]. The difference between NuMA and LGN position is unclear, whereas all signs imply their crucial role in the molecular mechanism underlie in the division orientation.

Another possible factor is the positioning of the centrosomes, because the centrosomes exist at each spindle pole as the organelles and microtubule organizing centre. After duplication, centrosomes separation could be achieved either by moving from one pole to the opposite one or by the simultaneous separation followed by the spindle rotation of into two poles of cell and then the bipolar spindle mitotic spindle are formed. As observed in *Drosophila* neuroblasts, one centrosome anchor at the cell cortex, the other migrates and then spindle orientation is accomplished, which implies that positioning of the spindle poles takes place before the mitosis [81–83]. However, experiments of immunostaining and by tracking fluorescently-labelled centrosomes have verified that centrosomes of basal cells in the epidermis positioning apically and pull the spindle into a perpendicular orientation during the early stages of mitosis, even as the mitotic spindle is forming [65,66,84]. Because positioning of centrosomes is not pre-determined prior to mitotic entry, the spindle of basal epidermal cell could align either laterally or perpendicularly.

### Oriented cell division and skin wound healing

As mentioned above, the skin is the protective, defensive barrier from the outside world. Once injured, the skin should restore the normal organizational morphology and physiological function efficiently. Wound healing biology refers to initiating relatively inactive cell lineages to the wound margin for the proliferation, invasion and reconstruction of new matrix in the wound gap [85]. Studies have illustrated that many people suffered from severe burns, occurrence of venous leg ulcer and chronic foot wounds from diabetes and the United States government cost very much for these [86–88]. Similarly, wounds in China are also severe problems and China has a huge demand for tissue repair and regeneration [89,90].

Wound healing is a complex event and consists of an inflammatory phase, re-epithelialization and remodelling phase [85,91–93]. At first, tissue injury disrupts vascular vessels and the release of growth factors, cytokines and components of ECM initiate inflammatory response. Then, tissue re-epithelialization is achieved through the migration and proliferation of keratinocytes to resurface the wound area with a layer of new epithelium. Finally, the collagen fibres reorganize and mature to gain tensile strength in the end. Generally speaking, skin injuries could be healed more quickly, but defective re-epithelialization may leave a connective tissue scar. During the re-epithelialization, keratinocytes at the wound edge conduct migration, proliferation and differentiation and reconstitute the epidermal integrity over the granulation tissue finally [94].

**Figure 3:**
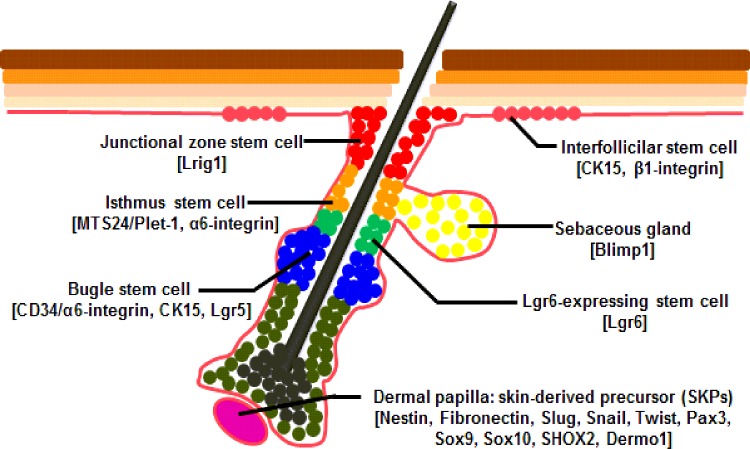
Location of SKPs and ESCs in the skin SKPs reside in dermal papilla (purple) and express nestin, fibronectin, Slug, Sail, Twist, Pax3, Sox9, Sox10, SHOX2 and Dermo1. ESCs have been identified to reside in different regions of skin, including the bulge (blue) expressed CD34/α6-integrin, CK15 and Lgr5, IFE (pink) expressed CK15 and β1-integrin, sebaceous gland progenitors (yellow) expressed Blimp1, the upper isthmus region (upper isthmus, orange), junctional zone between upper isthmus and IFE adjacent to the sebaceous glands of the HFs (red) expressed Lrig1 and a group of stem cells expressing Lgr6 has been found to locate directly above the follicle bulge (green).

It is reported that skin-derived precursor cells (SKPs) and ESCs are an important component in the process of skin wound healing [95–98], which are located in different regions in the skin and express specific molecular markers (Figure 3). SKPs are a novel population of neural crest-related precursor cells that mainly reside in dermal papilla and can be isolated from embryonic and adult skin. SKPs express proteins of nestin and fibronectin and also express a variety of neural crest (NC) associated transcription factors, including Slug, Snail, Twist, Pax3, Sox9, Sox10, SHOX2, Dermo1 [97,98]. The multiple populations of ESCs have been identified to reside in different locations with the skin. As a major reservoir for epidermal keratinocytes, the bulge region of the HFs expresses the following marker molecules: CD34/α6-integrin, CK15, leucine rich repeat containing G protein coupled receptor 5 (Lgr5) [99,100]. In conditions such as wounding, bulge stem cells rapidly migrate toward the upper bulge to help with the rapid regeneration of wounded skin and these progeny of slow cycling bulge cells express CK15 and β1-integrin in the IFE [101–103], placenta-expressed transcript 1 (MTS24/Plet-1) and α6-integrin isthmus region of the HFs [104], Blimp1 in the sebaceous gland [105], Lrig1 in the junctional zone between upper isthmus and IFE adjacent to the sebaceous glands of the HFs [106]. Additionally, resent study is reported that Lgr6 marked a group of stem cells directly above the follicle bulge can produce cells of both the HFs and the sebaceous gland during embryonic development and can be activated by wounding and migrated toward the epidermis for wound repair [107]. Studies have illustrated that ESCs play an important role in the re-epithelialization and remodelling stage during wound healing [108–110]. In general, ESCs undergo symmetric division to produce either two stem cells (symmetric, non-differentiative division) or two transient amplifying cells (symmetric, differentiative division). Although symmetric division could replace cells rapidly for healing wounds, inappropriate symmetric (differentiative) division may deplete stem cells and cause fibrosis, neurodegeneration, failure to repair damaged tissues, osteoporosis and degenerative changes associated with aging. Rather than proliferate symmetrically into two identical cells, asymmetric division can preserve the stabilization of the pool of the ESCs (Figure 2B). One of the daughter cells remains in a niche as a stem cell for future use, whereas the other daughter cell is transient amplifying cell for current use [111]. The transient amplifying cells undergo subsequent rounds of symmetric proliferative mitotic divisions and differentiate further into a specific lineage such as keratinocytes [110].

Therefore, oriented mitotic division of epithelial cells, especially ESCs, is particularly important for wound healing. The prolonged symmetric divisions of ESCs during early embryonic development generate large pools of stem cells for tissues repair in need. Perhaps the ability to switch back and forth between symmetric and asymmetric division, increases the capacity for repair and facilitates longer lifespan. When the cells undergo oriented mitotic division to repair the wound by natural process, the impaired skin can complete perfect repair; and when the cell division orientation is random, there will form fibre hyperplasia, abnormal tissue structure and eventually lead to scar formation. Up to now, the currently important questions are that the key factors decide the mitotic division of epithelial cells and how to regulate to achieve oriented cell division. As it should be, we can find some interesting clues from previous researches. But how to utilize these basic theories related cell mitotic division to promote wound healing is still a long way to go.

## PERSPECTIVES

Oriented cell divisions are crucial for a variety of developmental and homoeostatic processes ranging from elongation to stratification. Both PCP and Par/Insc pathway mainly control the oriented cell division in many different systems. However, many questions need to be resolved, such as mechanism controlled the position of the spindle, cortical determinants transmitting external cues to the actin cytoskeleton and microtubules, roles of cell–cell and cell–ECM interactions in determining how cells divide. A key issue for the future is how stem cells are regulated to switch between asymmetric and symmetric divisions in the development of tissues and organs and skin wound healing.

Furthermore, as the epidermis must maintain a balance of between asymmetric and symmetric divisions, it must also regulate the two types of division to ensure proper development. Then, how is the machinery required for asymmetric or symmetric cell division localized to specific regions in the cell? Whereas there have been significant advances in uncovering proteins involved in spindle orientation, we still do not have the full complement of players. Developmental questions are also outstanding. How does the epidermis commit to stratification? What are the developmental cues? This information may also shed light on how the balance of symmetric and asymmetric divisions is regulated to yield a normal sized and shaped skin. Maybe that is the key question to achieve perfectly wound repair in skin.

## Abbreviations

aPKC: atypical protein kinase C
Dctn1: dynactin 1
Dig: DIsplaced Gonad
Dlg: discs large
Dsh: dishevelled
ECM: extracellular matrix
ESC: epidermal stem cell
Fz: frizzled
GMC: ganglion mother cell
HF: hair follicle
IFE: interfollicular epidermis
Insc: inscuteable
Lgr5: leucine rich repeat containing G protein coupled receptor 5
mInsc: adaptor protein mInscuteable
MTS24/Plet-1: placenta-expressed transcript 1
Mud: mushroom body defect
NC: neural crest
NuMA: nuclear mitotic apparatus protein
Par: partitioning defective protein
PCP: planar cell polarity
Pins: partner of inscuteable
SKP: skin-derived precursor cell
SOP: sensory organ precursor
Stbm: strabismus
